# Illumina and PacBio DNA sequencing data, *de novo* assembly and annotation of the genome of *Aurantiochytrium limacinum* strain CCAP_4062/1

**DOI:** 10.1016/j.dib.2020.105729

**Published:** 2020-05-21

**Authors:** Christian Morabito, Riccardo Aiese Cigliano, Eric Maréchal, Fabrice Rébeillé, Alberto Amato

**Affiliations:** a Laboratoire de Physiologie Cellulaire Végétale, Université Grenoble Alpes, CEA, CNRS, INRAE, IRIG-LPCV, 38054 Grenoble Cedex 9, France; b Sequentia Biotech Carrer d'Àlaba, 61, 08005 Barcelona, Spain

**Keywords:** Genome, Third generation sequencing, Next generation sequencing, Structural annotation, Biotechnology, Thraustochytrid

## Abstract

The complete genome of the thraustochytrid *Aurantiochytrium limacinum* strain CCAP_4062/1 was sequenced using both Illumina Novaseq 6000 and third generation sequencing technology PacBio RSII in order to obtain trustworthy assembly and annotation. The reads from both platforms were combined at multiple levels in order to obtain a reliable assembly, then compared to the *A. limacinum* ATCC^Ⓡ^ MYA1381™ reference genome. The final assembly was annotated with the help of strain CCAP_4062/1 RNAseq data. *A. limacinum* strain CCAP_4062/1 is an industrial strain used for the production of very long chain polyunsaturated fatty acids, like the docosahexaenoic acid that is an essential fatty acid synthesised only at very low pace in humans and vertebrates . Thraustochytrids in general and *Aurantiochytrium* more specifically, are used for carotenoid and squalene production as well. Beside their biotechnological interest, thraustochytrids play a crucial role in both inshore and oceanic basins ecosystems. Genome sequences will foster biotechnological as well as ecological studies.

Specifications table

**Table:** 

**Subject**	Applied Microbiology and Biotechnology
**Specific subject area**	Marine eukaryotic microbiology
**Type of data**	DNA Sequencing Data
**How data were acquired**	The data were acquired by Next-Generation Sequencing technology using Illumina Novaseq 6000 and third generation sequencing technology using PacBio RSII platforms
**Data format**	Raw reads were deposited in GenBank. Analysed: The assembly (fasta file), the gene descriptions (txt file), and the gene models (gtf file) were deposited in Mendeley
**Parameters for data collection**	DNA was extracted from six day-old cultures.
**Description of data collection**	Whole-genome sequencing, genome assembly, and annotation
**Data source location**	Institution: LPCV-IRIG City: Grenoble Country: France *Aurantiochytrium limacinum* strain was collected in Mayotte (Indian Ocean, 12°48′51.8′’S, 45°14′21.7′’E)
**Data accessibility**	Repository name: NCBI BioProjects Data identification number: PRJNA612804 Direct URL to data: https://www.ncbi.nlm.nih.gov/bioproject/PRJNA612804 Repository name: Mendeley Data identification number: 10.17632/v3w485jnz5.2 Direct URL to data: http://dx.doi.org/10.17632/v3w485jnz5.2

## Value of the Data

• The biotechnology based on thraustochytrids has been gaining importance in the last decades. Physiology and life cycle traits can be investigated as well under a molecular point of view.
• The dataset presented here can provide information to both academic and private laboratories for reverse genetics studies.
• The genome can be advantageous for biotechnological as well as physiological studies aiming at improving growth or lipid production in thraustochytrids.

## 1 Data description

Thraustochytrids are marine non-photosynthetic protists whose ability to produce high amounts of lipids, like long-chain polyunsaturated fatty acids [1] used in nutraceutical, and some terpene derivatives [2] like astaxanthin, a potent antioxidant agent, and squalene [3], has attracted industrial interest [4]. Although ecologically relevant [5], *Aurantiochytrium limacinum* biotechnological attractiveness is the main trigger for transcriptomic and genomic studies. To date an increasing number of thraustochytrid genomes [6,7] and transcriptomes [8,9] have been sequenced, and this has fostered reverse genetics studies [10, 11]. Here we present the genome sequenced by Illumina and PacBio of an *A. limacinum* strain that groups in the same 18S rDNA clade as the type species of the genus *Aurantiochytrium* [1], *A. limacinum* strain SR21 stored at the American Type Culture Collection under the entry ATCC^Ⓡ^ MYA1381™. The taxonomic and systematic environment of thraustochytrids is very complex and requires profound rearrangements [12], but the phylogenetic position of strain CCAP_4062/1 was recently confirmed [1].

## 2 Genome assembly

The starting dataset included PacBio data obtained with the RSII long read technology and Illumina Novaseq 6000 reads (Table 1). The PacBio data included 770,000 polymerase reads with an N50 of 37,932 bp and a total of 12.16 Gbp. The Illumina data consisted of 59.9 million paired-end 2 × 150 bp reads corresponding to 9 Gbp [13]. Considering that the estimated genome size of *A. limacinum* is about 60 Mbp, the PacBio and Illumina data corresponded to a 202 × and 150 × coverage, respectively.

**Table 1:** Description of the genomics datasets used in this study.

	PacBio Sequel RSII	Illumina NovaSeq 6000
Sequenced Bases	12,160,726,429 bp	9,045,844,656 bp
Number of Reads	770,207	59,906,256
Sequencing Layout	Single End Long Reads	Paired End 2 × 150 bp
Max Read Length	120,000 bp	150 bp
Read N50	37,932 bp	150 bp
Estimate Genome Coverage	202 ×	150 ×

**Fig. 1:**
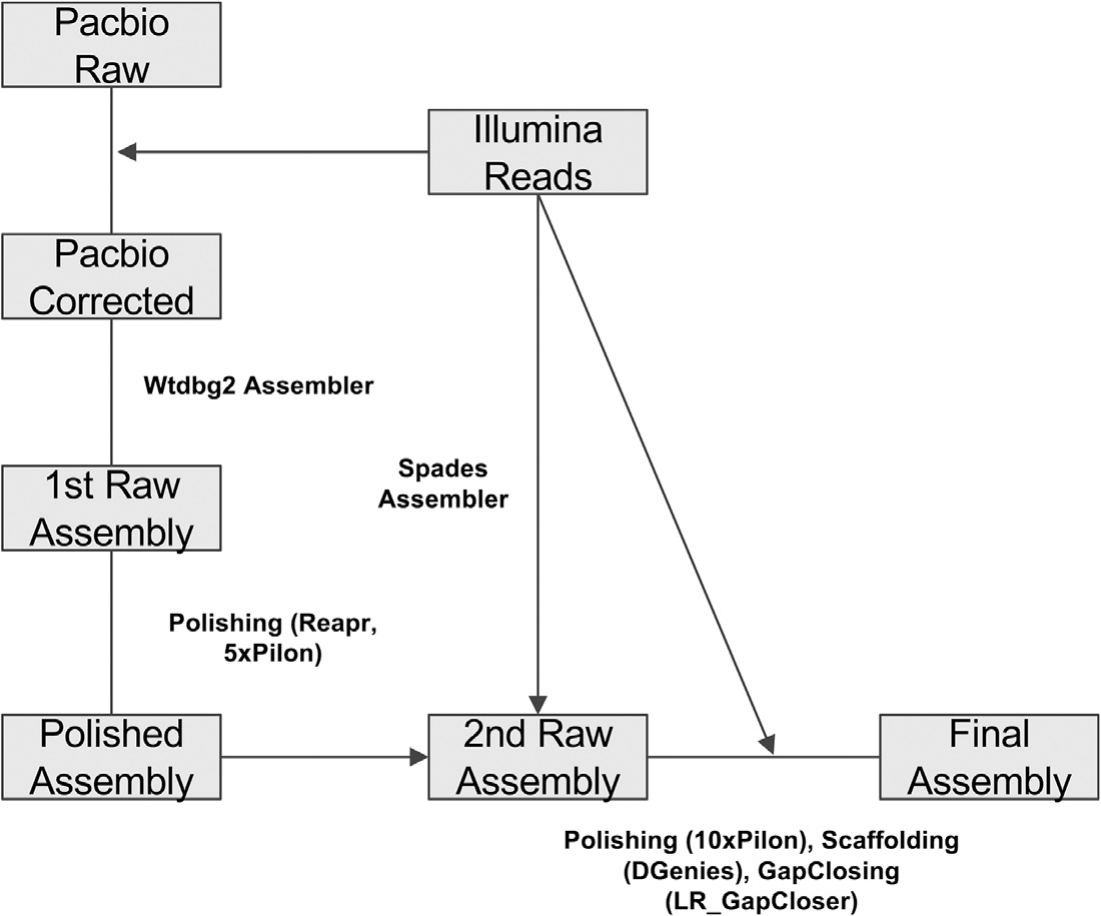
Schematic representation of the genome assembly pipeline. Raw PacBio reads were corrected using the Illumina data and using three iterations of the program LoRDEC. The corrected PacBio reads were used to create a draft assembly with the tool wtdbg2, thus producing the first raw assembly. The latter was then polished using the Illumina reads and performing five iterations of Pilon corrections and one run of REAPR to remove misassemblies. The polished assembly was used together with the Illumina reads to perform an assembly with Spades. The obtained assembly was polished with 10 iterations of Pilon, then gap closing was performed with LR_GapCloser.

In order to obtain a highly accurate genome assembly, the Illumina and PacBio datasets were combined at multiple levels following the bioinformatics pipeline showed in Fig. 1. The Illumina dataset was quality-checked and trimmed in order to remove adapters and low-quality sequences thus obtaining 47 million high quality reads. PacBio reads were corrected using the high-quality Illumina reads and used to perform a draft assembly with the software wtdbg2. The obtained genome sequence was polished using the Illumina reads and then used as a guide for a new assembly with Spades. The newly obtained genome was subjected to error correction, scaffolding and gap-closing. The procedure led to the final assembly.

**Fig. 2:**
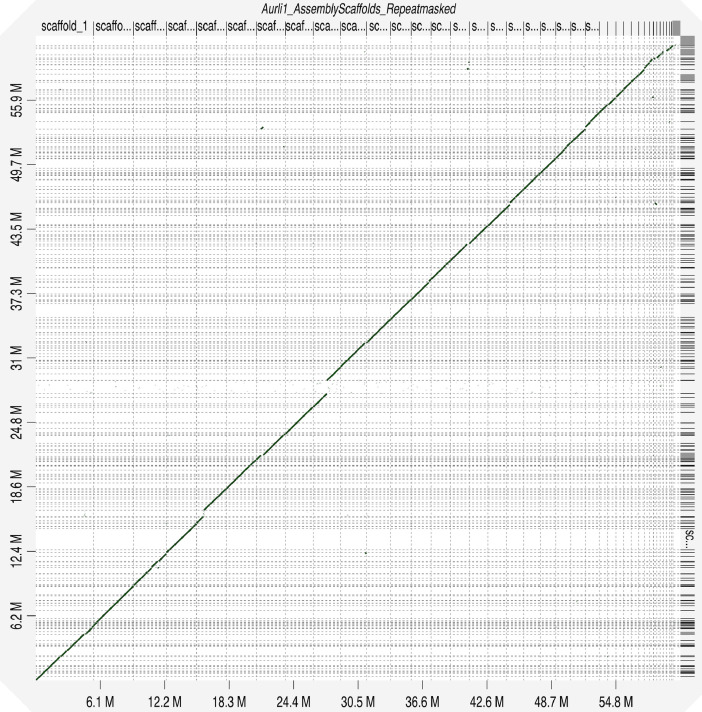
Dotplot obtained by aligning the Aurli1 reference genome [13] assembly from *A. limacinum* ATCC^Ⓡ^ MYA1381™ (*X*-axis) against the *Aurantiochytrium limacinum* strain CCAP_4062/1 scaffolds.

The obtained assembly was aligned against the *A. limacinum* ATCC^Ⓡ^ MYA1381™ reference genome [13] assembly as showed in Fig. 2. The analysis highlighted a high degree of collinearity between the two genomes with more than 93% of the sequences having an identity higher than 75%. Indeed, the Average Nucleotide Identity (ANI) between the two genomes was 98.89%.

Given the high collinearity and similarity of the two genomes, a scaffolding step was performed with the software DGenies using the *A. limacinum* ATCC^Ⓡ^ MYA1381™ assembly as a reference. The final assembly was then generated by creating a super scaffold with the smallest unplaced contig. The obtained final assembly included 478 sequences with a total assembled genome of 62 Mbp (Table 2).

**Table 2:** Genome assembly statistics.

	Aurantiochytrium limacinum strain CCAP_4062/1
Number of Contigs	478
Genome Size	62,086,374 bp
Number of Contigs larger than 50 Kbp	210
N50	358,008 bp
L50	51
Largest Contig	2029,424 bp
GC Content	45.66%

**Fig. 3:**
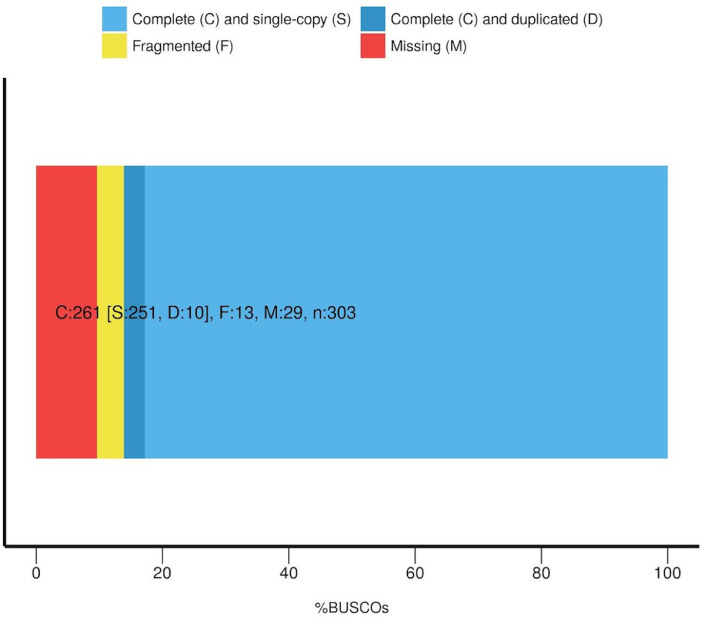
Results of the BUSCO analysis highlighting the presence of complete and single copy eukaryotic genes in the assembly. Letters indicate the BUSCO categories presented in the figure, numbers indicate the number of genes composing a category. ‘*n*’ indicate the total number of genes in all BUSCO categories.

The quality of the assembly was evaluated by mapping back the Illumina and PacBio reads to measure the percentage of alignment and its quality. About 97.7% of the Illumina reads were mapped back to the assembly with a mean mapping quality of 52 and 95% of the reads were properly paired. About 95% of the PacBio reads were mapped to the assembly with a mean mapping quality of 48. In addition, the tool BUSCO was used to predict the presence of single copy conserved Eukaryotic genes in the assembly (Fig. 3), highlighting that 86% of the genes were complete.

## 3 RNA-seq guided annotation of the genome

Once the final genome assembly was produced, genome annotation was performed. For this purpose, RNA-seq dataset [8] was used to assist in the gene prediction. The bioinformatics pipeline consisted in mapping the RNA-seq reads on the genome assembly followed by a combination of GeneMark and Augustus in order to predict the gene models. The Braker2 pipeline was used for this purpose. As a first step, the aligned RNA-seq reads were used to detect introns and transcribed loci. More than 95% of the reads could be correctly mapped on the obtained assembly. Then, the information used in the previous step was used to feed GeneMark to create an HMM model of splicing sites which, in turn, was used to train Augustus. In addition, the proteins from the *A. limacinum* ATCC^Ⓡ^ MYA1381™ genome were used as input to identify coding genes by sequence similarity. The generated Augustus model was used to create the final annotation. About 14,470 protein coding genes were predicted, of which 12,610 (87%) had a match with an *A. limacinum* ATCC^Ⓡ^ MYA1381™ protein. Gene descriptions and Gene Ontology annotations were associated to 11,208 proteins using the Pannzer 2 pipeline.

**Fig. 4:**
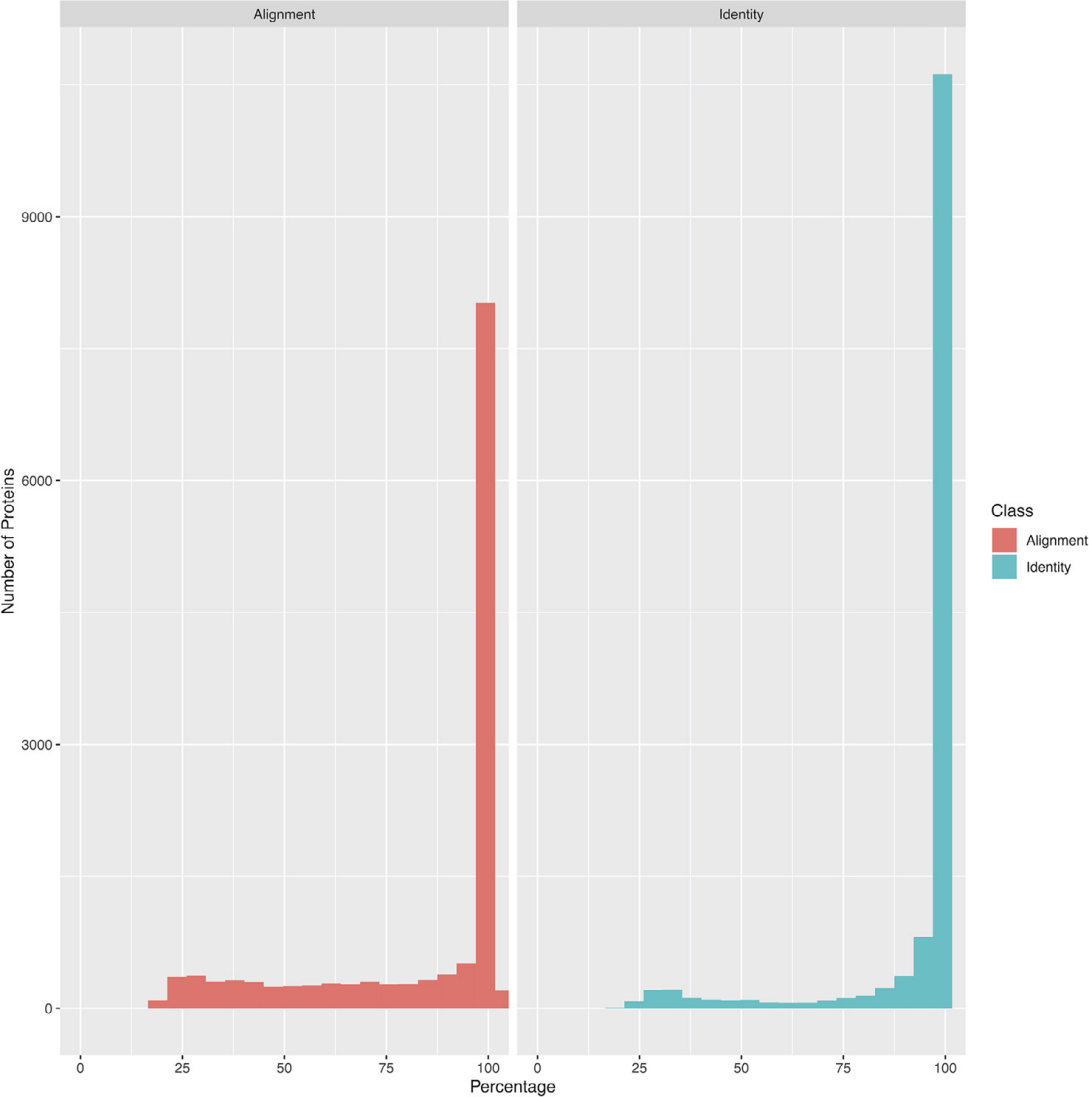
Histograms showing the distribution of alignment and identity percentage between *Aurantiochytrium limacinum* ATCC^Ⓡ^ MYA1381™ (reference) and *Aurantiochytrium limacinum* strain CCAP_4062/1 predicted proteins (present study).

To assess the quality of the annotation, the RNA-seq reads were used to detect the expression of the annotated genes and about 75% of the reads could be properly associated to the annotated genes. In addition, the length of the predicted proteins was compared with the length of *A. limacinum* ATCC^Ⓡ^ MYA1381™ proteins. About 90% of the predicted proteins covered at least 90% of the *A. limacinum* ATCC^Ⓡ^ MYA1381™ proteins, showing that the genes were complete (Fig. 4). Finally, a BUSCO analysis was performed on the predicted proteins using the Eukaryotic gene-set which highlighted the presence of 90% of complete genes, of which 87% were in single copy.

## 4 Experimental design, materials, and methods

### 4.1 The strain

By means of pollen grain bait method [14] carried out on samples from coastal seawater gathered in Mayotte (Indian Ocean, 12°48′51.8′’S, 45°14′21.7′’E), a cell was isolated and an axenic culture established. Once the culture was proved to be monospecific and axenic, it was deposited at the Culture Collection of Algae and Protozoa (CCAP) under the accession number CCAP_4062/1.

### 4.2 Culture conditions and DNA extraction

In order to accumulate biomass for DNA extraction, a six-day old culture was inoculated in 50 mL fresh R medium [1,5] at 5 × 10^5^ cells mL^−1^ in sterile 250 mL-Erlenmeyer glass flasks and incubated for six days at 20 °C and 100 rpm. Genomic DNA was extracted by phenol:chloroform:isoamyl alcohol (25:24:1) on lysed cells and precipitated with Na-Acetate 3 M pH 5 and absolute ethanol. Quality and concentration were estimated using a Thermo Scientific™ NanoDrop 2000 Spectrophotometer and Qubit Flex Fluorometer. Genomic DNA was sent to Macrogen (Korea) for both Illumina and PacBio sequencing. The sample was prepared according to a guide for preparing SMRTbell template for sequencing on the PacBio Sequel System. The templates were sequenced using SMRT Sequencing. Illumina TruSeq Nano DNA Kit was used to generate the Illumina library according to manufacturer's specifications. Illumina sequencing was performed on a Novaseq-6000 producing paired-end 2 × 150 bp reads.

### 4.3 Bioinformatics methods

Raw Illumina reads were analysed with FASTQC [15] to obtain quality statistics, then BBDuk v38.75 [16] was used to perform trimming and clipping (minimum base quality 35 and minimum read length 35 bp). PacBio reads were corrected using three iterations of the software LoRDEC v0.3 [17] together with the trimmed Illumina reads, the three iterations were performed with three different K-mer lengths: 19, 31 and 41 bp. The software wtdbg2 v2.5 [18] was used to obtain the first draft assembly with the PacBio corrected reads, an estimated genome size of 60 Mbp was indicated. The Illumina reads were then mapped against the obtained assembly with minimap2 version 2.17-r954-dirty [19] and the results treated with Pilon v1.23 [20] and REAPR v1.0.18 [21] in order to fix mismatches and assembly rearrangements. This process was iterated five times obtaining a polished first assembly. The obtained assembly was used as input, together with the Illumina reads, for Spades v3.14.0 [22] to perform a second genome assembly, which was then polished by 10 iterations of Pilon corrections. Gap closing was performed using the LR_GapCloser algorithm [23] using the polished first assembly as input. The obtained scaffolds were aligned with DGenies [24] against the *Aurantiochytrium limacinum* ATCC^Ⓡ^ MYA1381™ genome [13] using minimap2 as an aligner. The FASTA file containing the scaffolds ordered according to the *A. limacinum* ATCC^Ⓡ^ MYA1381™ genome alignment was finally downloaded. The CCAP_4062/1 scaffolds with no match against the *A. limacinum* ATCC^Ⓡ^ MYA1381™ genome were concatenated introducing 40 Ns between each scaffold to generate a FASTA of unplaced sequences. Genome assembly statistics were produced with the software QUAST [25] whereas BUSCO analyses were performed with the software version 3 [26] and the eukaryote_odb9 dataset. Average Nucleotide Identity between *A. limacinum* ATCC^Ⓡ^ MYA1381™ and *A. limacinum* strain CCAP_4062/1 genomes was calculated with FastANI [27].

For genome structural annotations, RNA-seq reads [8] were trimmed and clipped using BBDuk using the same parameters mentioned above. Reads were mapped against the genome assembly using STAR v2.7.3a [28] in double pass mode. The obtained mapping was used as input for Braker2 v2.1.0 [29] also providing a FASTA file containing the protein sequences of *A. limacinum* ATCC^Ⓡ^ MYA1381™. The genome assembly was repeat masked with RepeatMasker version open-4.0.9 [30] before performing the annotation selecting the option -species stramenopiles. Gene expression levels were obtained using Kallisto v0.46.0 [31] against the predicted transcript sequences. Finally, gene functional annotations were obtained using the software PANNZER2 [32] with the following options: Minimum query coverage 0.4 or minimum sbjct coverage 0.4, and Minimum alignment length 50. The ARGOT [33] scoring function implemented in PANNZER2 as default advanced option for Gene Ontology Annotation that proved to be the best [32], was chosen. The functional annotation table (available at doi:http://dx.doi.org/10.17632/v3w485jnz5.2) contains the description, Gene Ontology and KEGG Enzymes identified through PANNZER2. The four columns contain 1. **Locus**: The transcript ID created during the gene prediction step; 2. **Annotation Type**, which can include: DE (general description), MF_ARGOT (Gene Ontology Molecular Function), BP_ARGOT (Gene Ontology Biological Process), CC_ARGOT (Gene Ontology Cellular Component), EC_ARGOT (EC number); 3. **Annotation ID**, which contains the accession number in the case of MF_ARGOT, CC_ARGOT, BP_ARGOT and EC_ARGOT and the score of the prediction in the case of DE; 4. **Description** of the annotation ID, which includes the gene description in the case of DE, the Gene Ontology description for MF_ARGOT, CC_ARGOT, BP_ARGOT and the associated GO ID for the EC_ARGOT. The sequences of CCAP_4062/1 predicted proteins were aligned with the *A. limacinum* ATCC^Ⓡ^ MYA1381™ proteins using the BLASTp algorithm setting a minimum evalue of 0.001.

## Declaration of Competing Interest

The authors declare that they have no known competing financial interests or personal relationships which have, or could be perceived to have, influenced the work reported in this article.

## Appendix Supplementary materials

Image, application 1
